# Patterns of Detection and Capture Are Associated with Cohabiting Predators and Prey

**DOI:** 10.1371/journal.pone.0059846

**Published:** 2013-04-02

**Authors:** Billie T. Lazenby, Christopher R. Dickman

**Affiliations:** 1 School of Biological Sciences, University of Sydney, Sydney, New South Wales, Australia; 2 Department of Primary Industries Parks Water and Environment, Hobart, Tasmania, Australia; Australian Wildlife Conservancy, Australia

## Abstract

Avoidance behaviour can play an important role in structuring ecosystems but can be difficult to uncover and quantify. Remote cameras have great but as yet unrealized potential to uncover patterns arising from predatory, competitive or other interactions that structure animal communities by detecting species that are active at the same sites and recording their behaviours and times of activity. Here, we use multi-season, two-species occupancy models to test for evidence of interactions between introduced (feral cat *Felis catus*) and native predator (Tasmanian devil *Sarcophilus harrisii*) and predator and small mammal (swamp rat *Rattus lutreolus velutinus*) combinations at baited camera sites in the cool temperate forests of southern Tasmania. In addition, we investigate the capture rates of swamp rats in traps scented with feral cat and devil faecal odours. We observed that one species could reduce the probability of detecting another at a camera site. In particular, feral cats were detected less frequently at camera sites occupied by devils, whereas patterns of swamp rat detection associated with devils or feral cats varied with study site. Captures of swamp rats were not associated with odours on traps, although fewer captures tended to occur in traps scented with the faecal odour of feral cats. The observation that a native carnivorous marsupial, the Tasmanian devil, can suppress the detectability of an introduced eutherian predator, the feral cat, is consistent with a dominant predator – mesopredator relationship. Such a relationship has important implications for the interaction between feral cats and the lower trophic guilds that form their prey, especially if cat activity increases in places where devil populations are declining. More generally, population estimates derived from devices such as remote cameras need to acknowledge the potential for one species to change the detectability of another, and incorporate this in assessments of numbers and survival.

## Introduction

The interactions between animals can be important determinants of species’ distributions and abundances [1], [2], [3]. Observations of behaviour and experiments exploring animal responses to cues from co-occurring species have been an important source of information which has helped to describe and quantify relationships such as those between predators and between predators and prey (e.g., [4], [5], [6]).

Remote cameras are being used increasingly to study wildlife [7]. They can record multiple species and offer a unique opportunity to observe spatial and temporal patterns of visitation at camera sites by different taxa by time-stamping photographs, functioning for relatively long periods without user input compared to other methods such as sand padding, and operating in variable environmental conditions. Such data lend themselves to two-species occupancy modelling, which accounts for imperfect detection of different species within a maximum likelihood framework [8]. For example, if inverse spatial or temporal patterns between two species are observed, they may indicate that one species is dominant or inducing fear in the other. Asymmetrical interactions often occur between different-sized predators, predators and prey, or dominant and subordinate competitors [9], [10].

Two-species occupancy modelling is a robust method for modelling observations of patterns consistent with asymmetrical interactions; in addition to allowing for differences in detection probability between species, other covariates such as habitat, which can be important explanatory variables for observed patterns, can also be incorporated [8]. It is a good starting point for designing experiments that explicitly address the causal processes that determine patterns arising from interactions between species.

Fear, manifested as avoidance behaviour, is an important structuring component in many ecosystems [11], [12]. For example, small mammalian carnivores often fear larger species and alter their habitat use when dominants are present [13], [14], [15], leading to reduced population size and activity of the subordinate predators. Mesopredator release occurs when a subordinate predator responds to the decline or extinction of a dominant predator by increasing population size, activity, or impact on prey, and it has been observed in many ecosystems globally [16], [17]. The feral cat *Felis catus* L. provides a good example. This carnivore frequently occupies a mesopredator role and can have destructive impacts on small prey if the suppressive effects of a dominant species are removed [18], [19], [20], [21], [22].

Fear and avoidance also characterize many predator-prey relationships; in mammals, these relationships are often mediated by odour (e.g., [23]). Selection can be expected to favour individuals that recognize and avoid the odour of predators that are a significant source of mortality, hence reducing their probability of encounter with the predator itself [24], [25]. Avoidance of predator odours by small mammals is often pronounced where predators and prey have coevolved [4] but, in systems where predators are novel, avoidance responses by prey may not always occur (e.g., [26], [27]). Lack of recognition and avoidance of novel predators may explain, in part, why small mammals have fared poorly in Australia over the last 200 years [28]. Many species in the Australian region experience high rates of depredation from the recently introduced red fox *Vulpes vulpes* L. and feral cat, with at least 16 species declining to extinction in the presence of these predators [29]. However, some Australian rodents avoid the odours of novel predators, perhaps suggesting that predation already has selected for prey that can recognize and avoid them [30].

In this study we use cameras to uncover relationships between introduced and native predators and prey in Tasmania, a large (68 000 km^2^) island south of the continental mainland of Australia. Feral cats were originally introduced to Tasmania some 200 years ago [31], with red foxes arriving over the last decade or more [32], [33]. A sub-species of swamp rat, *Rattus lutreolus velutinus* (Thomas), is endemic to Tasmania and is particularly useful, as a potential prey species, for field-based behavioural studies because it is widespread and highly detectable compared to other native small mammals on the island. Adult swamp rats weigh on average 100 g [34] and have an approximately equal head-body compared to tail length, and ears that are set close to the head compared to those of co-occurring small mammals. These features make them distinguishable in photographs taken by remote cameras. The world’s largest extant marsupial carnivore, the Tasmanian devil *Sarcophilus harrisii* (Boitard), also is endemic to Tasmania [35]. There have been recent declines in devil populations of up to 85% in some areas as a result of a contagious and invariably fatal cancer – devil facial tumour disease (DFTD) [36]. These declines have been associated with increases in spotlight counts of feral cats, perhaps via mesopredator release [37]. Feral cats – at less than half the body mass of Tasmanian devils (adult devils weigh 8–10 kg whereas cats weigh 2–4 kg) – are highly likely to be subordinate in any encounters between the two species. As small mammals such as the swamp rat form a very large part of the diet of feral cats compared to devils [38], our goal was to better understand relationships between these predators and prey and improve our ability to manage them.

We investigated predator and prey interactions and responses to a range of predator odour cues in the cool temperate forests of southern Tasmania. Our specific aims were to 1) quantify patterns of detection at camera sites of devils and feral cats, devils and swamp rats, and feral cats and swamp rats; and 2) investigate the trap response of the swamp rat to devil and feral cat faecal odours. We hypothesized that feral cats would show reduced detection at camera sites where devils were recorded and that swamp rats would be detected less often at sites with devils and feral cats. We hypothesized further that swamp rats would avoid the faecal odours of feral cats and devils.

## Materials and Methods

### Ethics Statement

This study was conducted under ethics approval from the University of Sydney Animal Ethics Committee number L04/8/2008/3/4878 and scientific permits issued by the Tasmanian Department of Primary Industries Parks Water and Environment for research on native wildlife (TFA108121, TFA10046, and TFA11137).

### Study Sites

**Figure 1:**
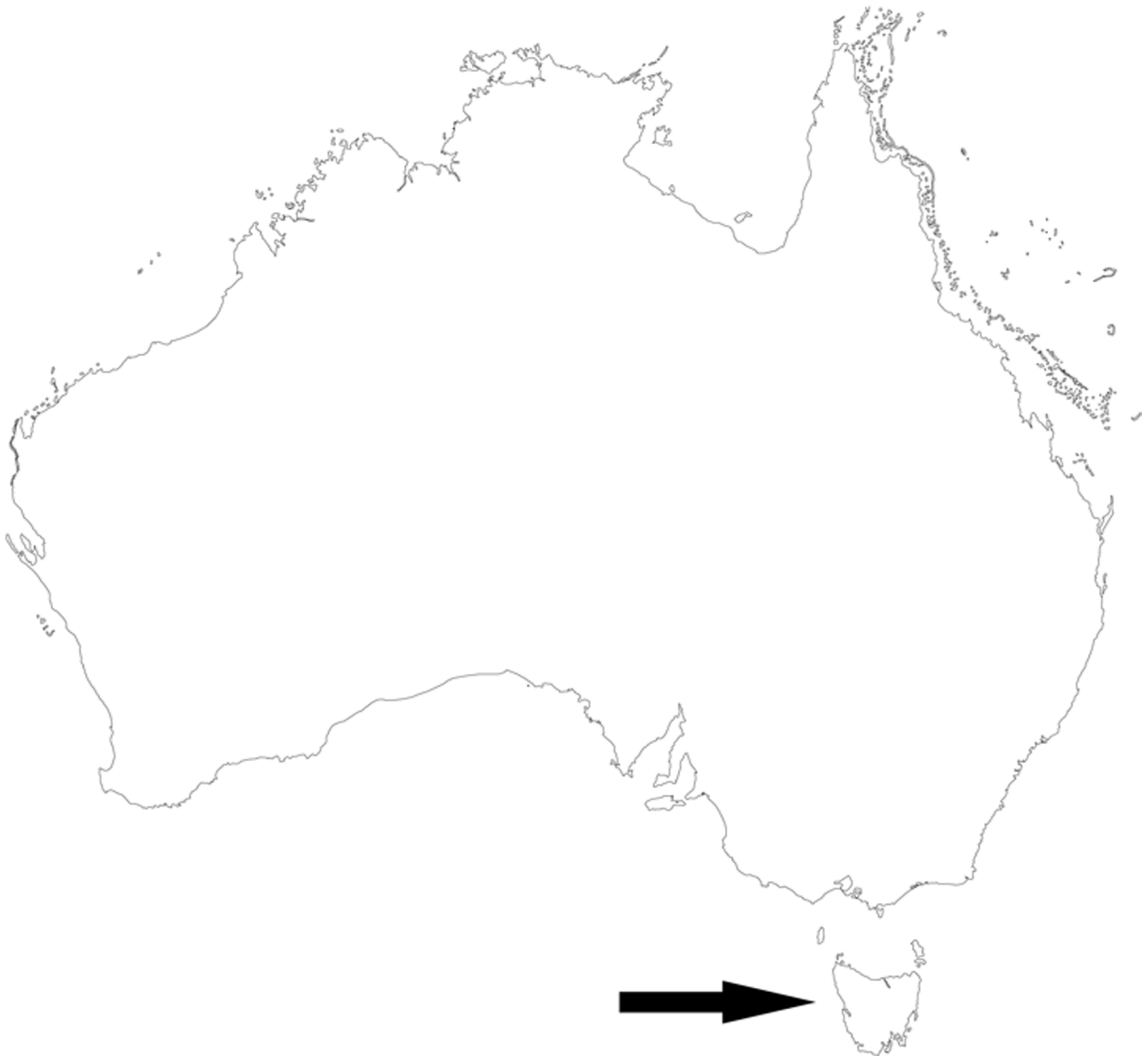
Map of Australia showing Tasmania. The arrow indicates the 68 000 km^2^ island State of Tasmania to the south of the continental mainland of Australia. Map courtesy of Geoscience Australia.

**Figure 2:**
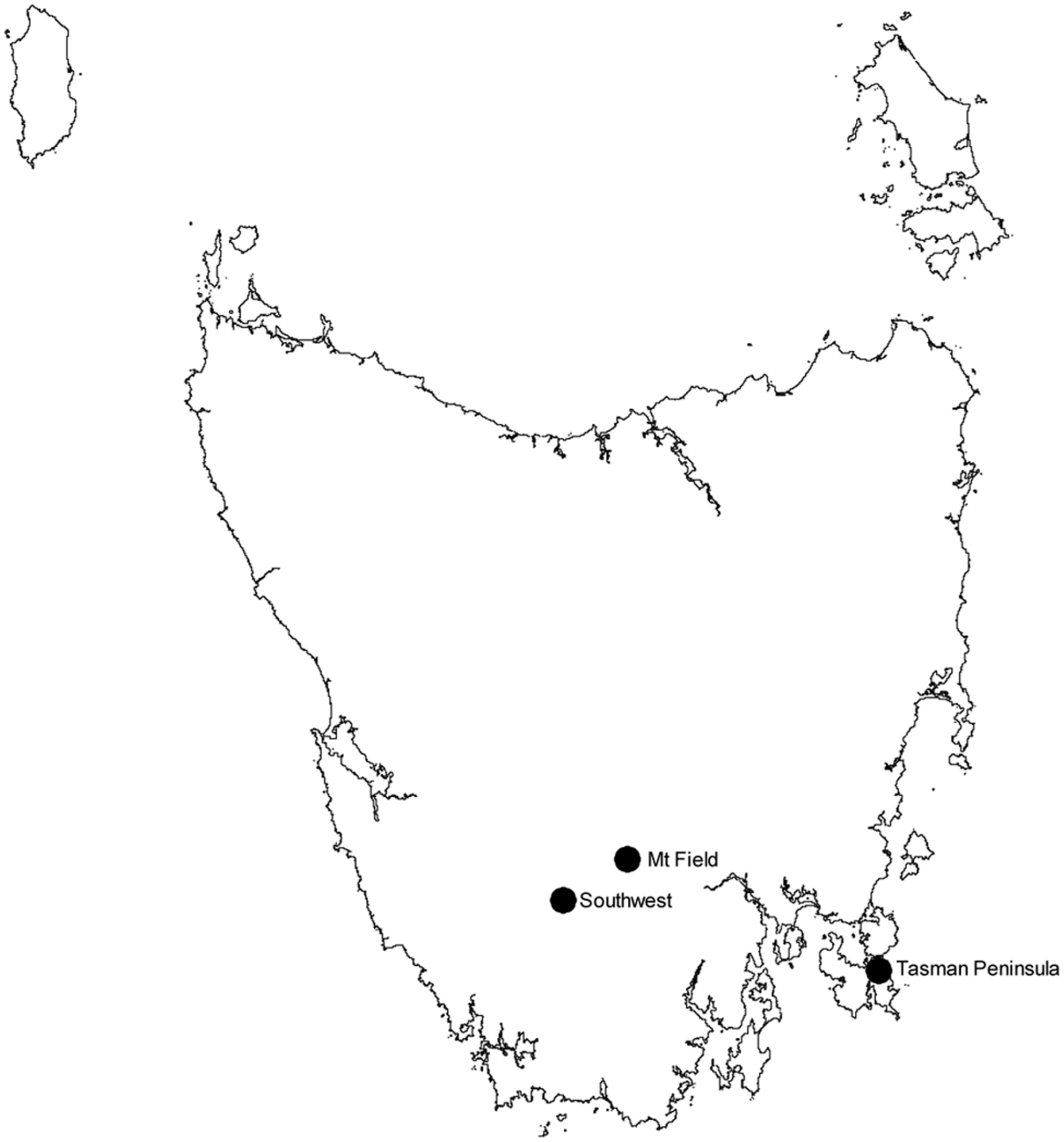
Map of Tasmania showing study sites. Each site is represented by a black dot.

Three study sites, each covering ∼40 km^2^, were selected in southern Tasmania based on similarities in flora, fauna and land use (Fig. 1). They are referred to henceforth as Southwest, Mt Field, and Tasman Peninsula (Fig. 2). The central coordinate for each site in longitude and latitude is: Southwest 146° 22′ 26.4″, −42° 48′ 28.8″; Mt Field 146° 41′ 16.8″, −42° 39′ 46.8″; and Tasman Peninsula 147° 54′ 36″, −43° 3′ 50.4″. The outer margins of each study site were separated by at least 15 km. Vegetation at each site was primarily cool temperate wet forest, but included minor components of other habitats such as highland treeless vegetation. More specifically, the vegetation dominating the three study sites, classified according to the TASVEG 2.0 Tasmanian mapping system [39], was:

- Dry eucalypt forest and woodland (emergent eucalypts over a relatively open ground storey compared to rainforest and wet eucalypt vegetation);
- Rainforest and related scrub (characterised by the following genera: *Nothofagus*, *Atherosperma*, *Eucryphia* and *Athrotaxis*);
- Wet eucalypt forest and woodland (emergent eucalypts over a rainforest or scrub community); and
- Agricultural exotic and urban vegetation (characterised by plantation operations including eucalypt and pine plantations). Plantations and active forestry operations occurred or had occurred recently in small parts of all study areas.

Areas that were not subjected to forestry operations were classified as ‘reserved land’ such as National Park. All study sites were traversed by a network of gravel roads, fire trails and other tracks.

The average annual maximum and minimum temperatures at the study sites, recorded by the Bureau of Meteorology, ranged from 7 to 16 degrees, and 1 to 9 degrees, respectively (www.bom.gov.au). Mean annual rainfall ranged from 960 to 2500 mm and there were above average rainfalls on all sites during 2009, 2010 and 2011.

### Remote Cameras

**Figure 3:**
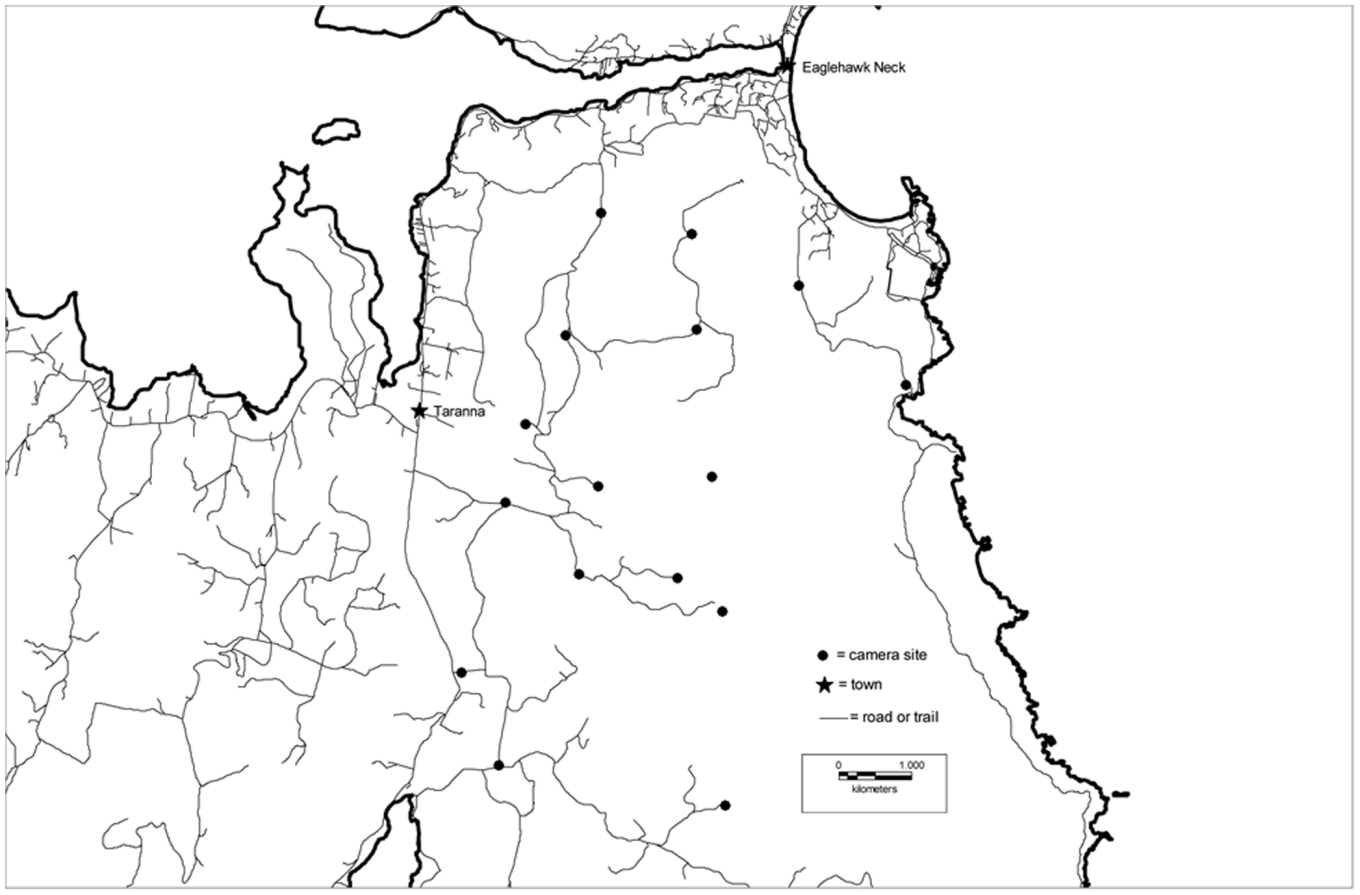
Map of camera placement within the Tasman Peninsula. An example of the spatial arrangement of cameras along roads and trails within a study site.

Up to 18 remote cameras (a minimum of 15 and an average of 17) were set for at least one week per survey at each study site in April and June 2009 and at least two weeks per survey in April and again in June at each site in 2010 and 2011. We used DigitalEye™ 7.2 (Pixcontroller Inc.) digital trail cameras set 1.0–1.5 km apart on the sides of dirt and gravel vehicular trails. They were set in a systematic grid pattern and placed in the same locations for each survey (Fig. 3). All units were set 5–10 m from a track or trail in one of the four vegetation types (Table 1) to hide them from public view.

**Table 1:** Vegetation associated with camera sites.

Study area	Dry eucalypt forest and woodland	Rainforest and related scrub	Wet eucalypt forest and woodland	Agricultural exotic and urban vegetation
Southwest	4	0	5	8
Mt Field	2	8	2	6
Tasman Peninsula	3	3	0	11

The number of camera sites situated within different vegetation types within each study area.

The cameras featured an infra-red flash that is less likely to disturb animals compared to visible white light flashes [40]. They also had a passive infra-red triggering system that detects body heat and motion before triggering a photo. Each remote camera consisted of a removable 7.2 megapixel digital camera, electronics control board and 9 V battery, all encased in a waterproof, camouflage-painted pelican case. We set the passive infrared sensitivity (PIR) switch at medium (standard factory setting), and the switch control board to record in ‘trail mode’ so that photographs were taken at least once every second after the PIR sensor had been activated by an animal. The switch control board was also set such that the camera would record photographs 24 hours a day.

Cameras were fixed to solid tree trunks ∼30 cm above ground using straps. They were set in positions that received full shade throughout the day to reduce the contrast in light and shadow on the ground; this reduced triggering of the PIR sensor and avoided runs of empty photographs. Cameras were tilted 5 to 10 degrees from the vertical by wedging the back of the camera unit to limit the PIR sensor range to an area that could be illuminated by the infra-red flash.

A scent lure and food reward consisting of Juro™ tuna emulsion (Juro Oz Pro tackle, Australia) and fish-based tinned cat food in jelly was placed 1.5–2.0 m from each camera unit for each survey. Two dessert spoons of tinned cat food and 50–75 ml of tuna emulsion were spread in a 0.25 m^2^ area that was the focal point for the camera. Tuna emulsion was also squirted on 1–2 branches up to 2 m off the ground above the focal point of the camera to maximize the chances of the lure scent entering air streams. Additional tuna emulsion was placed in a perforated film canister that was staked into the ground after the first two standard camera surveys. Mid-trip checks were conducted during surveys lasting more than a week (i.e. all the 2010 and 2011 surveys) to re-bait, and to replace camera batteries and memory cards. Photographs from remote cameras were downloaded on completion of each survey. The time-stamp on each photograph was converted to Eastern Standard Time.

#### Storage of information

**Figure 4:**
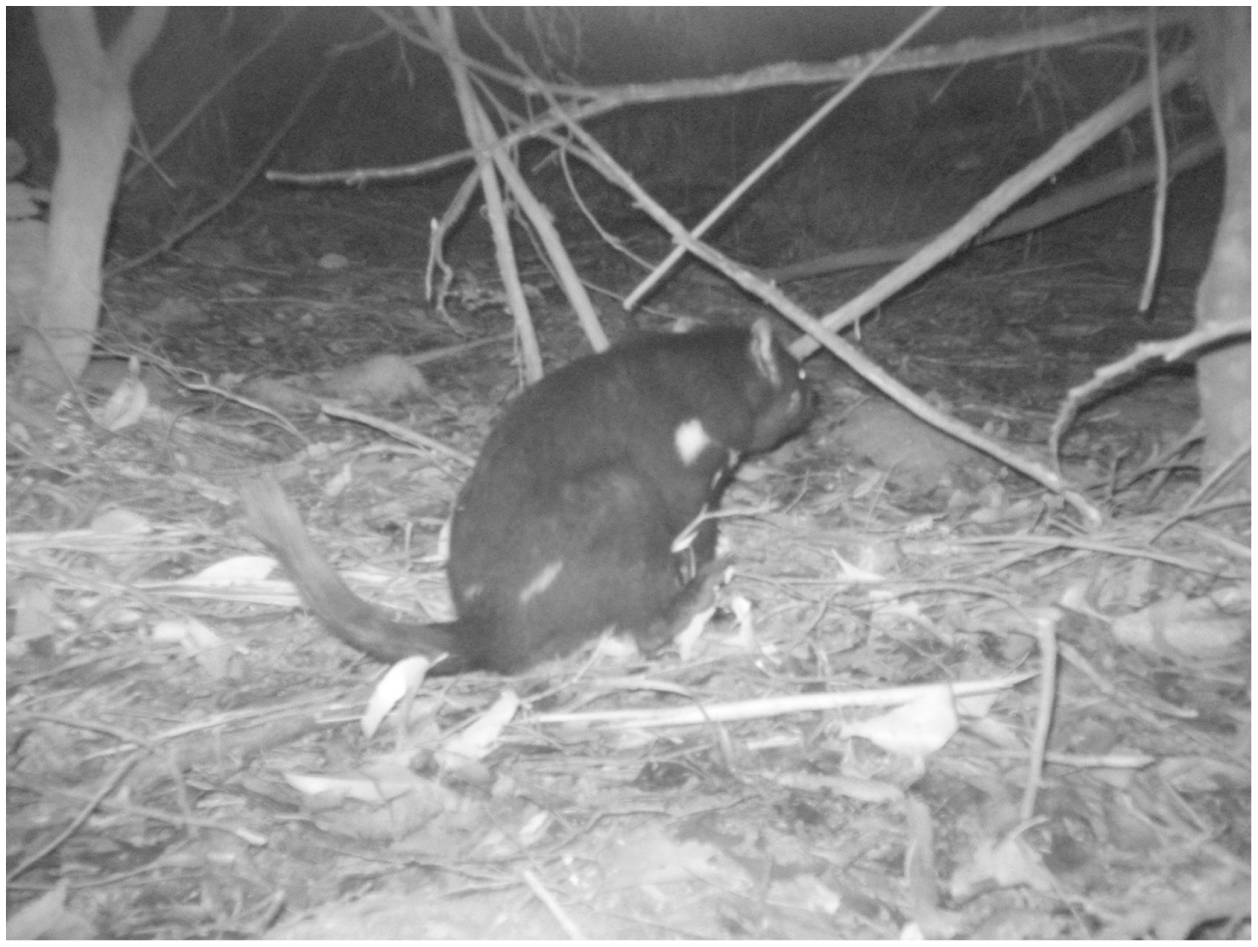
A Tasmanian devil *Sarcophilus harrisii*, scent-marking a camera station with an anal drag. Note the curved tail, raised hind foot, and posterior part of the body touching the ground. Scent-marking was often observed in a series of photographs and was evidenced by anal-dragging, defecating or urinating.

We stored information from over 58 500 photographs collected during the study in a Microsoft AccessTM database. This included time, date, species, confidence of species identification, 24 h survey period (from 1700 h to 1700 h the following day), study site and camera site. We also recorded whether sites were scent marked by devils (Fig. 4). Behaviours consistent with scent-marking were clearly distinguishable in photographs and included anal dragging, defecating and urinating.

We used a query that allowed us to extract occurrence matrices for each species and each survey (custom written by Studio Q, Sandy Bay, Tasmania). The matrices consisted of columns for 24 h survey periods and rows for camera numbers. A ‘1’, ‘0’ or ‘.’ was recorded in each cell where ‘1’ signified one or more occurrences within the defined 24 h period, ‘0’ signified no recorded occurrences, and ‘.’ denoted camera failure.

### Small Mammal Trap Response to Predator Odours

Faecal odour trials were conducted at two of the three study sites in April and June 2011 using type-A (30×10×10 cm) Elliott small mammal traps (Elliott Scientific, Upwey, Victoria). Trials were carried out away from cameras to avoid any interference. We trialed four odours: feral cat, Tasmanian devil, Tasmanian pademelon *Thylogale billardierii* (Desmarest), and clear (no scent). Faecal odours of the herbivorous pademelon were included to ensure that any avoidance by small mammals of predator-scented traps represented genuine avoidance rather than a simple preference for clean traps.

Fresh faecal samples were obtained from a variety of wild and captive sources and then frozen. Carnivore scats were placed in a freezer at −80°C for at least two weeks to kill pathogens such as *Toxoplasma gondii* and hydatid tapeworms, after which time they were stored in a normal commercial freezer at −20°C. A portion of each scat was removed and placed in a container labeled with the species of origin to defrost prior to deployment. Where possible, we included portions of scat from different individuals and sexes in defrosting containers. Following defrosting, each container was mixed into a faecal slurry with water (ratio ∼5∶1), with care taken not to cross-contaminate the odours of different species.

We constructed a grid of 20 trap stations and set four Elliott traps at each station. Stations were separated by 20–30 m. A smear of slurry was placed on the door of each Elliott trap (excluding the ‘clear’ odour) such that there was one of each of the scented traps at each trapping station. Traps labeled with their respective scents were set in a circular pattern, ∼1 m apart, opening towards the centre of the circle. This protocol follows those used in several previous investigations [25], [26], [28].

Traps were baited with a mixture of peanut butter, rolled oats and honey; a handful of commercial rabbit bedding (clean woodchip shavings with the dust extracted) also was placed at the back of each trap for insulation. A waterproof plastic sheet was placed over the back of the traps and secured to keep captured animals dry. Traps were left *in situ* for four nights, but were re-scented after two nights. Trapping stints of a maximum of four nights were separated by at least a month before faecal odour trials were carried out at the same sites. Faecal odour trials were conducted over a total of 13 nights. Traps were checked from first light every morning, and captured animals were weighed, measured, marked with an individual-specific ear clip, then released. Traps that captured animals were replaced with clean traps with new bait, bedding and fresh faecal smears. Dirty traps were scrubbed with water and air dried in sunlight off-site. Records from trap stations capturing more than one animal overnight were excluded from analyses as animals captured after the first-caught individual would experience less choice of trap odours. Captures of the same individual over successive days were included.

### Analyses

#### Camera surveys

We used two-species multi-season occupancy models to test whether the presence of one species altered the probability of detection of another at cameras [8], [41]. We standardized camera survey effort by restricting our analyses to the first 7 or 14 days for 2009 and 2010–2011 respectively. Models were constructed in PRESENCE 4.0 [42] for the following pairs of species: devils and feral cats; devils and swamp rats; and feral cats and swamp rats. The models were based on data from over 3570 camera trap nights. We limited our analyses to patterns of detection because our camera spacing was not distant enough to ensure spatial independence between survey units for the two larger species; the devil and feral cat, therefore comparisons of probability of site occupancy may have produced erroneous results [38].

Two-species models contain many parameters, and large data sets are necessary to achieve reliable model convergence and variance estimates. As a result, the only small mammal included in our modelling was the swamp rat because of its frequent occurrence across the study sites. We considered modelling small mammals as a group, but preliminary investigation [38] revealed that different small mammal species had different habitat associations and probabilities of detection, thus rendering them unsuitable to be treated as one group.

Three alternative set-ups can be implemented in PRESENCE 4.0 for two-species multi-season occupancy models [41]. We used parameterization number one, which is conceptually the simplest alternative. We did not experience problems with model convergence that are sometimes associated with covariates in this parameterization, probably because we limited our analyses to detection rather than occupancy and detection. Parameterization one consists of the following parameters:

PsiA - the probability of occupancy for species A, regardless of occupancy status of species B

PsiB - the probability of occupancy for species B, regardless of occupancy status of species A

pA - the probability of detecting species A during the jth survey, if only species A is present

pB - the probability of detecting species B during the jth survey, if only species B is present

rA - the probability of detecting species A, given both species are present

rB – the probability of detecting species B, given both species are present

Phi – an expression of whether two species co-occur independently at survey sites; i.e. a ‘species interaction factor’ (SIF); it is defined by the following equation:
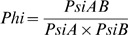
where PsiAB = the probability of both species being present.

Values <1 indicate that two species co-occur less often than expected, suggesting possible avoidance or competitive exclusion, while values >1 indicate a positive association.

Delta – an expression of whether two species are detected independently at survey sites, and termed a ‘detection species interaction factor’; it is defined by the following equation:
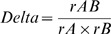
where rAB = the probability of detecting both species.

Values <1 indicate that cameras are less likely to detect one species during a 24 h survey period if the other species was detected during the same 24 h period, and the converse if values are >1.

Gamma – the probability that an unoccupied site in season t is occupied by the species in season t+1.

Epsilon - the probability that a site occupied in season t is unoccupied by the species in season t+1.

Given that our analyses were limited to probability of detection, parameters PsiA and PsiB were modelled independently, Phi was not estimated, and gamma and epsilon were modelled as constants.

Several environmental variables, considered *a priori* to have the potential to influence probabilities of detection at camera sites, were included in the two-species models. These were:

Study area – a covariate for each of the three study sites. Study sites where one of the species in the two-species model was recorded rarely were excluded from the analyses. This included Mt Field for the devil and swamp rat model, and Mt Field for the feral cat and swamp rat model.

Time since baiting – a score for the number of days elapsed since camera sites had been baited, such that bait was modelled to decay linearly (i.e. the first survey day was scored 0, the second 0.1, the third 0.2 and so on) over each 7-day period.

Habitat – classification of camera sites into one of four vegetation groups based on groupings in the TASVEG 2.0 Tasmanian vegetation mapping system [39]. The habitat groups were dry eucalypt forest and woodland, wet eucalypt forest and woodland, rainforest and related scrub, and agricultural, exotic and urban vegetation. There was a generally similar representation by habitat groups at camera sites across the three study areas (Table 1). Vegetation structure and cover can influence species’ habitat preferences and hence occupancy and detection probabilities; however, we used broad classifications of habitat based largely on floristics rather than microhabitat assessments of structure and cover. This was because selection of camera sites within habitat groups was standardized to facilitate better camera function towards microhabitats that received day-long shade and were clear of vegetation within the sensitivity range of the camera.

Season – a covariate for seasonal variation in detection probability. We were unable to model season with other covariates because the resulting models had a large number of parameters for our data set (30 parameters for a global model of season alone). Multi-season single-species site occupancy models indicated that there was little seasonal variation in detection probability for devils and feral cats but there was more seasonal variation for swamp rats [38].

Several questions were addressed regarding the probability of detection of the tested species at camera sites:

Was detection independent, irrespective of the species present within each 24 h period; i.e. did delta = 1?

Did the presence of one species at a camera site during a one or two week study site survey affect the detection of another; i.e. did pA = rA and/or pB = rB?

Models were ranked using Akaike’s Information Criterion (AIC). Models with small AIC values (delta AIC ≤2) were considered reasonable descriptors of the data, and the above hypotheses were tested by setting various constraints on the top-ranked model such as delta to equal one, pA to equal rA, and pB to equal rB.

#### Faecal odour trials

Numbers of single captures in traps bearing each odour-type were tallied over both study areas, and differences in capture frequencies tested using χ^2^ goodness of fit analyses.

## Results

**Figure 5:**
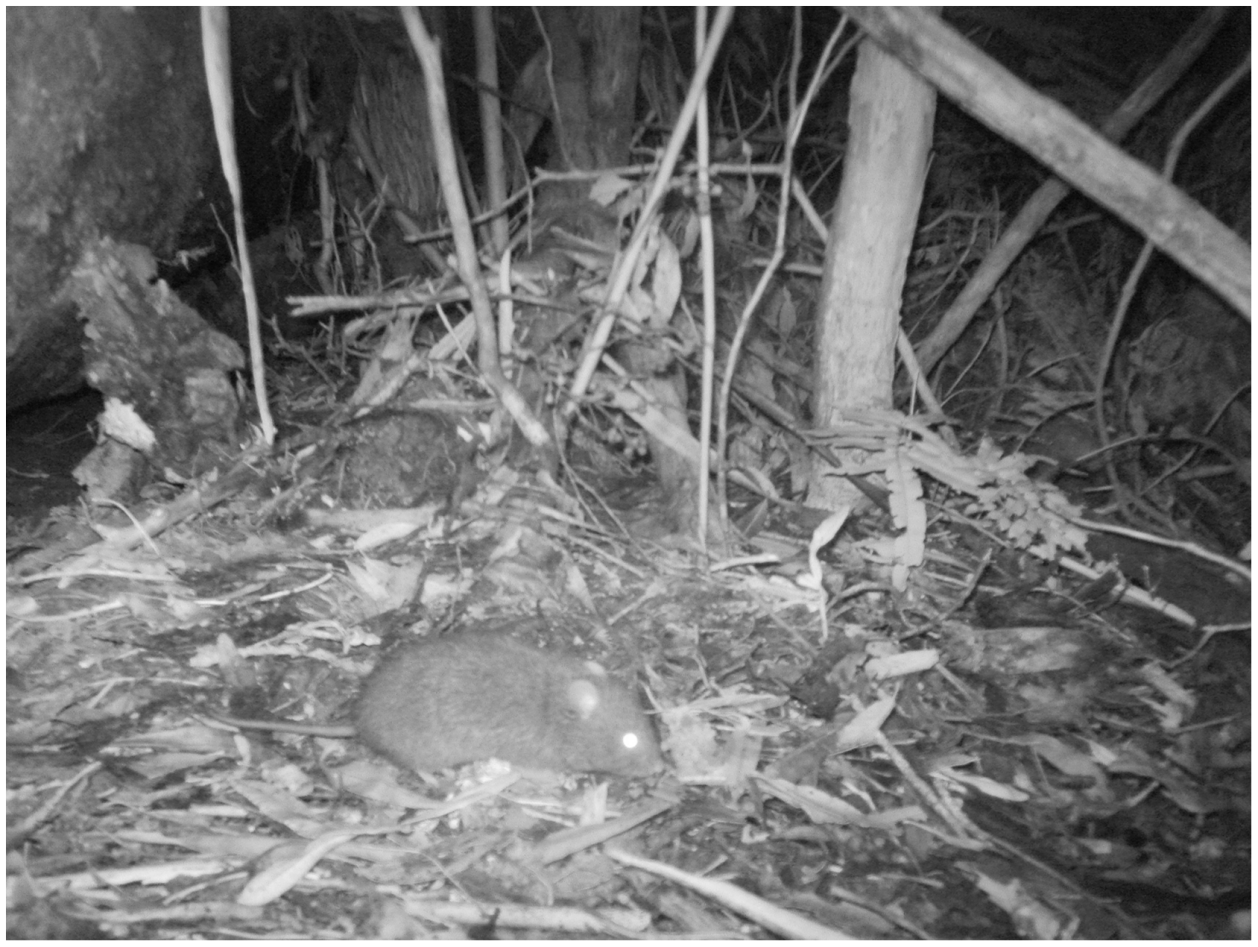
A swamp rat, *Rattus lutreolus velutinus*, visiting a camera station. Note the distinctive equal head-body compared to tail length, and ears that are set close to the head.

We recorded at least 23 species of mammals from 3754 visits at camera sites, as well as another 985 visits by birds (Table 2). Fifty-nine observations were made of devils scent marking during the course of the study between 2009 and 2011; 21 in Southwest, 32 at Mt Field, and six on the Tasman Peninsula (Fig. 4). There were 389, 308 and 249 records at cameras within 24 h periods of the devil, swamp rat (Fig. 5), and feral cat respectively. These data formed the basis of the two-species occupancy models. There were no photographs of the introduced red fox.

**Table 2:** Animal occurrences at cameras.

Species or group	Common name	Small, medium or carnivore*	No. of occurrences	Southwest	Mt Field	Tasman Peninsula
*Rattus lutreolus*	Swamp rat	Small	753 (308)	316 (143)	17 (12)	420 (153)
*Thylogale billardierii*	Tasmanian pademelon	Medium	617	98	114	405
*Mus musculus*	House mouse	Small	409	0	83	326
*Sarcophilus harrisii*	Tasmanian devil	Carnivore	389 (290)	164 (126)	192 (138)	33 (26)
*Trichosurus vulpecula*	Brush-tailed possum	Medium	329	27	394	59
*Dasyurus viverrinus*	Eastern quoll	Carnivore	281	3	278	0
*Pseudomys higginsi*	Long-tailed mouse	Small	258	111	17	130
*Felis catus*	Feral cat	Carnivore	249 (187)	75 (58)	65 (45)	109 (84)
*Rattus rattus*	Black rat	Small	211	0	51	160
*Dasyurus maculatus*	Spotted-tailed quoll	Carnivore	55	54	0	1
*Antechinus swainsonii*	Dusky antechinus	Small	45	1	7	37
*Potorous tridactylus*	Long-nosed potoroo	Medium	33	7	0	26
*Macropus rufogriseus*	Bennett’s wallaby	Medium	32	1	24	7
*Vombatus ursinus*	Wombat	Medium	26	6	5	15
*Canis familiaris*	Dog	Other	15	0	1	14
*Tachyglossus aculeatus*	Echidna	Medium	15	2	6	7
*Bettongia gaimardi*	Tasmanian bettong	Medium	8	0	8	0
*Perameles gunnii*	Eastern Barred Bandicoot	Medium	9	0	4	5
*Isoodon obesulus*	Southern Brown Bandicoot	Medium	8	3	3	2
*Hydromys chrysogaster*	Water rat	Small	3	3	0	0
*Pseudocheirus peregrinus*	Ringtail Possum	Medium	3	2	0	1
*Sminthopsis leucopus*	White-footed dunnart	Small	2	2	0	0
*Cercartetus* sp.	Pygmy possum	Small	1	1	0	0
Total small mammals			2281	1136	272	873
Unidentified small mammals			599	216	97	286
Total medium mammals			1399	170	612	617
Unidentified medium mammals (including carnivores)			165	24	55	86
Total carnivores			974	296	535	143
Total ground-foraging birds			985	428	190	367

The total numbers of occurrences (expressed as visits to a camera station – each visit with no longer than 5 minutes duration between consecutive photographs) and number of occurrences per study area (Southwest, Mt Field and Tasman Peninsula) for species, and animal groups, at camera sites set for 14 nights in 2009 and 28 nights in 2010 and 2011 in cool temperate forest in southern Tasmania. Numbers in parentheses represent the number of detections per 24 h survey period for species used in two-species occupancy models.

* Mammals were classified as small if the average adult weight of the species was 1–499 g, or medium for 500 g or above. The carnivore group included all medium to large mammalian carnivores such as feral cats, Tasmanian devils, eastern quolls and spotted-tailed quolls. It did not include domestic dogs, which were included in a separate category.

### Two-Species Models

#### Devils and feral cats

Two-species models comparing site occupancy and detection probabilities of devils and feral cats at camera sites are shown in Table 3, and the outputs from the models within two AIC points of the top model are shown in Table 6. The results (derived from models within two AIC points of the top model) indicate that:

**Table 3:** Model selection statistics for Tasmanian devils and feral cats.

Model	AIC	delta AIC*	AIC wt†	Model likelihood	No. par‡	−2xLogLik**
p*Sh* (s+h), p*Fc*(bxs), r*Sh* (s+h),r*Fc* (bxs),delta(.)	3059.11	0.00	0.3801	1.0000	22	3015.11
p*Sh* (s), p*Fc*(bxs), r*Sh*(s),r*Fc* (bxs),delta(.)	3059.20	0.09	0.3633	0.9560	19	3021.20
p*Sh* (bxs), p*Fc* (bxs), r*Sh*(bxs),r*Fc* (bxs),delta(.)	3061.94	2.83	0.0923	0.2429	21	3019.94
p*Sh* (s), p*Fc*(bxs), r*Sh*(bxs),r*Fc*(s),delta(b)	3061.94	2.83	0.0923	0.2429	20	3021.94
p*Sh* (s+h), p*Fc* = r*Fc* (bxs),r*Sh* (s+h),delta(.)	3063.97	4.86	0.0335	0.0880	18	3027.97
p*Sh* (s), p*Fc* (bxs), rSh(s),rFc (bxs),delta(s)	3065.69	6.58	0.0142	0.0373	21	3023.69
p*Sh* (bxs+h), p*Fc* (bxs+h), r*Sh*(bxs+h),rFc (bxs+h),delta(.)	3066.90	7.79	0.0077	0.0203	24	3018.90
p*Sh* (s), p*Fc = *r*Fc* (bxs), r*Sh*(s),delta(.)	3067.09	7.98	0.0070	0.0185	15	3037.09
p*Sh* (s), p*Fc*(s), r*Sh*(s),r*Fc* (s),delta(.)	3067.34	8.23	0.0062	0.0163	17	3033.34
p*Sh* (bxs), p*Fc = *r*Fc* (bxs), r*Sh*(bxs),delta(.)	3069.12	10.01	0.0025	0.0067	17	3035.12
**p** ***Sh*** ** = r** ***Sh*** ** (s+h), p** ***Fc*** **(bxs),r** ***Fc*** ** (bxs),delta(.)**	**3073.58**	**14.47**	**0.0003**	**0.0007**	**19**	**3035.58**
**p** ***Sh*** ** = r** ***Sh*** ** (s), p** ***Fc*** **(bxs),r** ***Fc*** ** (bxs),delta(.)**	**3077.29**	**18.18**	**0.0000**	**0.0001**	**16**	**3045.29**
**p** ***Sh*** **(s+h), p** ***Fc*** **(bxs), r** ***Sh*** ** (s+h), r** ***Fc*** ** (bxs),delta( = 1)**	**3083.09**	**23.98**	**0.0000**	**0.0000**	**22**	**3039.09**
**p** ***Sh*** **(s), p** ***Fc*** **(bxs), r** ***Sh*** ** (s), r** ***Fc*** ** (bxs),delta( = 1)**	**3085.50**	**26.39**	**0.0000**	**0.0000**	**19**	**3047.50**

The ten most supported multi-season two-species occupancy models based on AIC (Akaike’s Information Criterion) in program PRESENCE 4.0. Models that did not fall within the top ten that tested specific hypotheses are shown in bold below the top ten. The models were fitted to detection data from three study sites in cool temperate forests in southern Tasmania during standardized surveys from 2009–2011. The terms in parentheses represent the sources of variation in model parameters. ‘s’ denotes study site (Southwest, Mt Field and Tasman Peninsula), ‘h’ denotes habitat (dry eucalypt forest and woodland, wet eucalyptus forest and woodland, rainforest and related scrub, and agricultural exotic), ‘b’ denotes days since baiting, and ‘season’ denotes a model where detection varied with season. ‘.’ indicates a parameter set equal across species and survey times. The probabilities of site occupancy of devils and feral cats were estimated independently, and the probability of site occupancy, colonization and extinction were constrained to be constant for both species and covariates for all models. Tasmanian devil *Sarcophilus harrisii* is abbreviated *Sh* and feral cat *Felis catus* are abbreviated *Fc*.

Delta AIC is the difference in AIC values between each model and the model with the lowest AIC.

† AIC wt is the model weight.

‡ Number of parameters in the model.

** Twice the negative log-likelihood.

**Figure 6:**
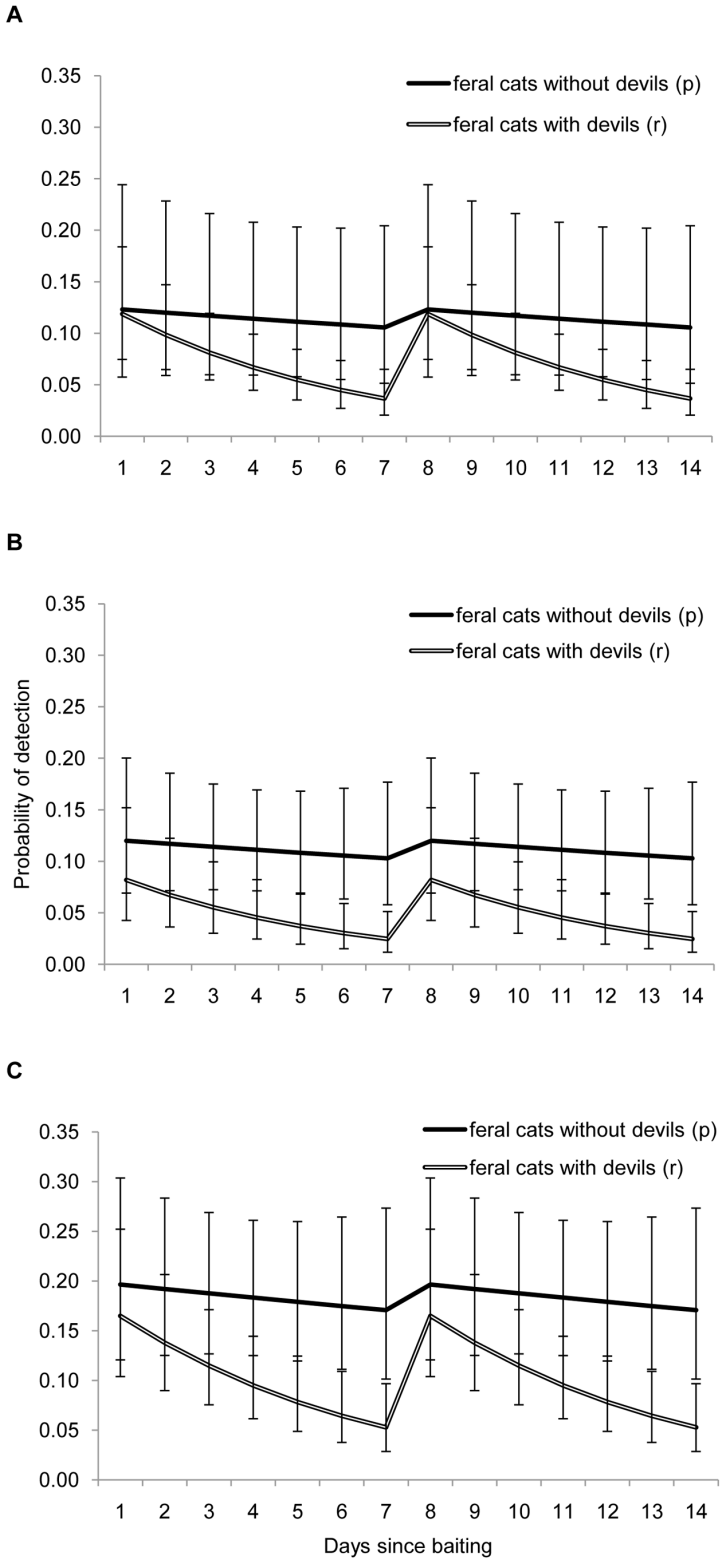
The probability of detecting feral cats *Felis catus* in relation to Tasmanian devils *Sarcophilus harrisii.* Study sites represented are: A. Southwest, B. Mt Field and C. Tasman Peninsula. Trends are shown as a function of time since baiting; cameras were initially baited on day one and re-baited on day seven. Estimates of probability of detection were generated in PRESENCE 4.0 under the highest-ranked AIC_c_ model in a two-species multi-season analysis. Bars represent 95% CI.

- Devils had a lower probability of site occupancy compared to feral cats; the probability of devil detection also varied between study sites, being markedly low on the Tasman Peninsula.
- Feral cats were detected consistently less often at cameras where devils were detected (Fig. 6). This pattern was consistent across the three study sites that were tested, and the probability of detecting a feral cat at sites where devils were detected was often less than half that of sites where devils were not detected (Figs 6A,B,C).
- Devils were detected more often at cameras where feral cats had been detected. Mt Field was an exception; here, devils were detected at similar rates regardless of the detection of feral cats.
- Feral cats and devils were detected less often than expected if their detections were independent within the same 24 h period. In other words, the probability of detecting both cats and devils within the same 24 h period was less than the probability of detecting either species alone. Estimates of delta under the most supported models ranged from 0.46 to 0.47 (±0.07 SE).
- Habitat and study site were important covariates for devil detection. Baiting and site were important covariates for feral cat detection.

#### Devils and swamp rats

Models within two AIC points of the top model comparing site occupancy and detection probabilities of devils and swamp rats (Table 4, Table 7) indicate that:

**Table 4:** Model selection statistics for Tasmanian devils and swamp rats.

Model	AIC	delta AIC*	AIC wt†	Model likelihood	No. par‡	−2xLogLik**
p*Sh* (s+h), p*Rl*(bxs+h), r*Sh*(s+h),r*Rl* (bxs+h),delta(bxs)	2314.33	0.00	0.2654	1.0000	20	2274.33
p*Sh* = r*Sh* (s+h), p*Rl*(bxs+h),r*Rl* (bxs+h),delta(bxs)	2314.46	0.13	0.2487	0.9371	18	2278.46
p*Sh* = r*Sh* (s+h), p*Rl*(bxs+h),r*Rl* (bxs+h),delta(s)	2316.20	2.01	0.1042	0.3962	17	2282.20
p*Sh* = r*Sh* (xbxs+h),p*Rl* (xbxs+h),r*Rl* (xbxs+h),delta(xbxs)	2316.45	2.12	0.0919	0.3465	19	2278.45
p*Sh* (bxs), p*Rl*(bxs), r*Sh*(bxs),r*Rl* (bxs),delta(bxs)	2317.63	3.30	0.0510	0.1920	19	2279.63
p*Sh* = r*Sh* (xbxs),p*Rl* (xbxs),r*Rl* (xbxs+h),delta(xbxs)	2317.81	3.48	0.0466	0.1755	16	2285.81
p*Sh* = r*Sh* (xbxs+h),p*Rl* (xbxs+h),r*Rl* (xbxs+h),delta( = 1)	2318.68	4.35	0.0301	0.1136	17	2284.68
p*Sh* (bxs), p*Rl*(bxs), r*Sh*(bxs),r*Rl* (bxs),delta(b)	2318.80	4.47	0.0284	0.1070	18	2282.80
p*Sh* (xbxs+h),p*Rl* (xbxs+h), r*Sh* (xbxs+h),r*Rl* (xbxs+h),delta(xbxs)	2319.37	5.04	0.0214	0.0805	22	2275.37
p*Sh* (xbxs+h),p*Rl* (xbxs+h), r*Sh* (xbxs+h),r*Rl* (xbxs+h),delta(xbxs+h)	2319.39	5.06	0.0211	0.0797	22	2275.39
**p** ***Sh*** ** (s+h), p** ***Rl*** **(bxs+h), r** ***Sh*** **(s+h),r** ***Rl*** ** (bxs+h),delta(bxs)**	**2320.67**	**6.34**	**0.0111**	**0.0420**	**18**	**2284.67**
**p** ***Sh*** ** (s+h), p** ***Rl*** ** = r** ***Rl*** ** (bxs+h), r** ***Sh*** **(s+h),delta(bxs)**	**2345.14**	**30.81**	**0.0000**	**0.0000**	**17**	**2311.14**

The ten most supported multi-season two-species occupancy models based on AIC (Akaike’s Information Criterion) in program PRESENCE 4.0. Models that did not fall within the top ten that tested specific hypotheses are shown in bold below the top ten. The models were fitted to detection data from two study sites in cool temperate forests in southern Tasmania during standardized surveys from 2009–2011. The terms in parentheses represent the sources of variation in model parameters. ‘s’ denotes study site (Southwest and Tasman Peninsula), ‘h’ denotes habitat (dry eucalypt forest and woodland, wet eucalyptus forest and woodland, rainforest and related scrub, and agricultural exotic), ‘b’ denotes days since baiting, and ‘season’ denotes a model where detection varied with season. ‘.’ indicates a parameter set equal across species and survey times. The probabilities of site occupancy of devils and swamp rats were estimated independently, and the probability of site occupancy, colonization and extinction were constrained to be constant for both species and covariates for all models. Tasmanian devil *Sarcophilus harrisii* is abbreviated *Sh* and swamp rat *Rattus lutreolus* is abbreviated *Rl*.

Delta AIC is the difference in AIC values between each model and the model with the lowest AIC.

† AIC wt is the model weight.

‡ Number of parameters in the model.

** Twice the negative log-likelihood.

**Table 5:** Model selection statistics for feral cats and swamp rats.

Model	AIC	delta AIC*	AIC wt†	Model likelihood	No. par‡	−2xLogLik**
p*Fc* (bxs),p*Rl* (bxs+h),r*Fc* (bxs),r*Rl* (bxs+h),delta (s)	2271.95	0	0.6358	1	21	2229.95
p*Fc* (bxs),p*Rl* (bxs+h), r*Fc* (bxs),r*Rl* (bxs+h),delta(bxs)	2273.94	1.99	0.2351	0.3697	22	2229.94
p*Fc* (bxs),p*Rl* (bxs+h),r*Fc* (bxs),r*Rl* (bxs+h),delta(.)	2275.52	3.57	0.1067	0.1678	20	2235.52
p*Fc* (bxs+h),p*Rl* (bxs+h), r*Fc* (bxs+h),r*Rl* (bxs+h),delta(bxs+h)	2281.12	9.17	0.0065	0.0102	22	2237.12
p*Fc* (bxs),p*Rl* (bxs+h),r*Fc* (bxs),r*Rl* (bxs+h),delta( = 1), gam,eps	2281.17	9.22	0.0063	0.01	20	2241.17
p*Fc* (bxs),p*Rl* (bxs), r*Fc* (bxs),r*Rl* (bxs),delta(bxs), gam,eps	2282.82	10.87	0.0028	0.0044	19	2244.82
p*Fc* (bxs),p*Rl* (bxs+h), r*Fc* (bxs),r*Rl* (bxs+h),delta(bxs+h), gam,eps	2282.86	10.91	0.0027	0.0043	22	2238.86
p*Fc* = r*Fc* (bxs),p*Rl* (bxs+h),r*Rl* (bxs+h),delta(s), gam,eps	2283.26	11.31	0.0022	0.0035	18	2247.26
p*Fc* (bxs),p*Rl* (bxs),r*Fc* (bxs),r*Rl* (bxs),delta(.), gam,eps	2284.78	12.83	0.001	0.0016	17	2250.78
p*Fc* (bxs),p*Rl* (bxs+h),r*Fc* (bxs),r*Rl* (bxs+h),delta(b), gam,eps	2285.22	13.27	0.0008	0.0013	21	2243.22
**p** ***Fc*** ** (bxs),p** ***Rl*** ** = r** ***Rl*** ** (bxs+h),r** ***Fc*** ** (bxs),delta(s)**	**2305.43**	**33.48**	**0**	**0**	**18**	**2269.43**

The ten most supported multi-season two-species occupancy models based on AIC (Akaike’s Information Criterion) in program PRESENCE 4.0. Models that did not fall within the top ten that tested specific hypotheses are shown in bold below the top ten. The models were fitted to detection data from two study sites in cool temperate forests in southern Tasmania during standardized surveys from 2009–2011. The terms in parentheses represent the sources of variation in model parameters. ‘s’ denotes study site (Southwest and Tasman Peninsula), ‘h’ denotes habitat (dry eucalypt forest and woodland, wet eucalyptus forest and woodland, rainforest and related scrub, and agricultural exotic), ‘b’ denotes days since baiting, and ‘season’ denotes a model where detection varied with season. ‘.’ indicates a parameter set equal across species and survey times. The probabilities of site occupancy of feral cats and swamp rats were estimated independently, and the probability of site occupancy, colonization and extinction were constrained to be constant for both species and covariates for all models. Feral cat *Felis catus* is abbreviated *Fc* and swamp rat *Rattus lutreolus* is abbreviated *Rl*.

* Delta AIC is the difference in AIC values between each model and the model with the lowest AIC.

† AIC wt is the model weight.

‡ Number of parameters in the model.

** Twice the negative log-likelihood.

**Table 6:** Model outputs from two-species occupancy models for Tasmanian devils and feral cats.

p*Sh* (s+h), p*Fc*(bxs), r*Sh* (s+h),r*Fc* (bxs),delta(.)							
Psi *Sh*	Psi *Fc*	p *Sh*	p *Fc*	r *Sh*	r *Fc*	delta	
0.63 (0.12)	0.70 (0.13)	SW 0.07 (0.02) D	SW 0.12 (0.05) 1	SW 0.13 (0.03) D	SW 0.12 (0.03) 1	0.47 (0.07)	
		SW 0.09 (0.03) W	SW 0.12 (0.04) 2	SW 0.18 (0.03) W	SW 0.10 (0.02) 2		
		SW 0.10 (0.03) R	SW 0.11 (0.04) 3	SW 0.19 (0.03) R	SW 0.08 (0.01) 3		
		MtF 0.17 (0.06) R	SW 0.11 (0.04) 4	MtF 0.15 (0.05) R	SW 0.07 (0.01) 4		
		MtF 0.12 (0.05) D	SW 0.11 (0.04) 5	MtF 0.10 (0.03) D	SW 0.05 (0.01) 5		
		MtF 0.17 (0.04) W	SW 0.11 (0.04) 6	MtF 0.14 (0.03) W	SW 0.05 (0.01) 6		
		MtF 0.28 (0.04) A	SW 0.11 (0.04) 7	MtF 0.24 (0.04) A	SW 0.04 (0.01) 7		
		TP 0.002 (0.002) D	MtF 0.12 (0.03) 1	TP 0.05 (0.02) D	MtF 0.08 (0.02) 1		
		TP 0.005 (0.004) A	MtF 0.12 (0.03) 2	TP 0.13 (0.04) A	MtF 0.07 (0.02) 2		
		TP 0.002 (0.002) W	MtF 0.11 (0.03) 3	TP 0.07 (0.02) W	MtF 0.06 (0.02) 3		
			MtF 0.11 (0.02) 4		MtF 0.05 (0.01) 4		
			MtF 0.11 (0.03) 5		MtF 0.04 (0.01) 5		
			MtF 0.11 (0.03) 6		MtF 0.03 (0.01) 6		
			MtF 0.10 (0.03) 7		MtF 0.02 (0.01) 7		
			TP 0.20 (0.05) 1		TP 0.17 (0.04) 1		
			TP 0.19 (0.04) 2		TP 0.14 (0.03) 2		
			TP 0.19 (0.04) 3		TP 0.11 (0.02) 3		
			TP 0.18 (0.03) 4		TP 0.10 (0.02) 4		
			TP 0.18 (0.04) 5		TP 0.08 (0.02) 5		
			TP 0.17 (0.04) 6		TP 0.06 (0.02) 6		
			TP 0.17 (0.04) 7		TP 0.05 (0.02) 7		
**p** ***Sh*** ** (s), p** ***Fc*** **(bxs), r** ***Sh*** **(s),r** ***Fc*** ** (bxs),delta(.)**							
**Psi ** ***Sh***	**Psi ** ***Fc***	**p ** ***Sh***	**p ** ***Fc***	**r ** ***Sh***	**r ** ***Fc***	**delta**	
0.62 (0.12)	0.76 (0.12)	SW 0.08 (0.03)	SW 0.12 (0.05) 1	SW 0.17 (0.02)	SW 0.11 (0.03) 1	0.46 (0.07)	
		MtF 0.08 (0.03)	SW 0.12 (0.04) 2	MtF 0.26 (0.02)	SW 0.09 (0.02) 2		
		TP 0.002 (0.005)	SW 0.11 (0.04) 3	TP 0.07 (0.02)	SW 0.08 (0.02) 3		
			SW 0.11 (0.04) 4		SW 0.07 (0.01) 4		
			SW 0.10 (0.04) 5		SW 0.05 (0.01) 5		
			SW 0.10 (0.04) 6		SW 0.05 (0.01) 6		
			SW 0.10 (0.03) 7		SW 0.04 (0.01) 7		
			MtF 0.12 (0.03) 1		MtF 0.06 (0.02) 1		
			MtF 0.12 (0.03) 2		MtF 0.05 (0.01) 2		
			MtF 0.11 (0.02) 3		MtF 0.04 (0.01) 3		
			MtF 0.11 (0.02) 4		MtF 0.03 (0.01) 4		
			MtF 0.11 (0.02) 5		MtF 0.03 (0.01) 5		
			MtF 0.10 (0.03) 6		MtF 0.02 (0.01) 6		
			MtF 0.10 (0.03) 7		MtF 0.02 (0.01) 7		
			TP 0.21 (0.06) 1		TP 0.15 (0.04) 1		
			TP 0.20 (0.05) 2		TP 0.13 (0.03) 2		
			TP 0.20 (0.04) 3		TP 0.11 (0.02) 3		
			TP 0.19 (0.04) 4		TP 0.09 (0.02) 4		
			TP 0.18 (0.04) 5		TP 0.08 (0.02) 5		
			TP 0.18 (0.04) 6		TP 0.06 (0.02) 6		
			TP 0.17 (0.04) 7		TP 0.05 (0.02) 7		

Outputs are shown for models that were within two AIC of the most supported model. ‘s’ represents study site (SW = Southwest, MtF = Mt Field or TP = Tasman Peninsula), ‘h’ represents habitat (D = dry eucalypt forest and woodland, W = wet eucalypt forest and woodland, R = rainforest, A = agricultural and/or exotic vegetation), ‘b’ represents days since baiting (1 = less than one day since baiting, 2 = less than two days since baiting and so on) and ‘.’ represents constant. Numbers in brackets represent standard error.

**Table 7:** Model outputs from two-species occupancy models for Tasmanian devils and swamp rats.

p*Sh* (s+h), p*Rl*(bxs+h), r*Sh*(s+h),r*Rl* (bxs+h),delta(bxs)						
Psi *Sh*	Psi *Rl*	p *Sh*	p *Rl*	r *Sh*	r *Rl*	delta
0.99 (0.17)	0.11 (0.06)	SW 0.16 (0.03) D	SW 0.21 (0.10) D 1	SW 0.16 (0.03) D	SW 0.46 (0.05) D 1	SW 0.66 (0.06) 1
		SW 0.15 (0.02) W	SW 0.17 (0.08) D 2	SW 0.15 (0.02) W	SW 0.44 (0.05) D 2	SW 0.57 (0.05) 2
		SW 0.11 (0.02) R	SW 0.13 (0.07) D 3	SW 0.11 (0.02) R	SW 0.41 (0.04) D 3	SW 0.47 (0.05) 3
		TP 0.04 (0.01) D	SW 0.11 (0.06) D 4	TP 0.05 (0.01) D	SW 0.40 (0.04) D 4	SW 0.38 (0.06) 4
		TP 0.02 (0.01) A	SW 0.08 (0.05) D 5	TP 0.02 (0.01) A	SW 0.36 (0.04) D 5	SW 0.29 (0.08) 5
		TP 0.04 (0.01) W	SW 0.06 (0.04) D 6	TP 0.04 (0.01) W	SW 0.34 (0.04) D 6	SW 0.22 (0.08) 6
			SW 0.05 (0.03) D 7		SW 0.32 (0.05) D 7	SW 0.16 (0.08) 7
			TP 0.78 (0.07) D 1		TP 0.26 (0.05) D 1	TP 0.58 (0.15) 1
			TP 0.73 (0.07) D 2		TP 0.24 (0.05) D 2	TP 0.48 (0.15) 2
			TP 0.67 (0.07) D 3		TP 0.22 (0.04) D 3	TP 0.39 (0.15) 3
			TP 0.61 (0.06) D 4		TP 0.21 (0.04) D 4	TP 0.30 (0.14) 4
			TP 0.54 (0.06) D 5		TP 0.19 (0.04) D 5	TP 0.23 (0.13) 5
			TP 0.47 (0.07) D 6		TP 0.17 (0.04) D 6	TP 0.17 (0.11) 6
			TP 0.40 (0.08) D 7		TP 0.16 (0.04) D 7	TP 0.12 (0.09) 7
**p** ***Sh*** ** = r** ***Sh*** ** (s+h), p** ***Rl*** **(bxs+h),r** ***Rl*** ** (bxs+h),delta(bxs)**						
**Psi ** ***Sh***	**Psi ** ***Rl***	**p ** ***Sh***	**p ** ***Rl***	**r ** ***Sh***	**r ** ***Rl***	**delta**
0.74 (0.16)	0.11 (0.06)	SW 0.15 (0.02) D	SW 0.60 (0.11) 1	SW 0.15 (0.02) D	SW 0.37 (0.05) 1	SW 0.66 (0.07) 1
		SW 0.16 (0.02) W	SW 0.55 (0.11) 2	SW 0.16 (0.02) W	SW 0.35 (0.05) 2	SW 0.58 (0.06) 2
		SW 0.12 (0.02) R	SW 0.51 (0.11) 3	SW 0.12 (0.02) R	SW 0.32 (0.04) 3	SW 0.50 (0.05) 3
		TP 0.04 (0.01) D	SW 0.46 (0.10) 4	TP 0.04 (0.01) D	SW 0.29 (0.04) 4	SW 0.42 (0.07) 4
		TP 0.02 (0.01) A	SW 0.41 (0.10) 5	TP 0.02 (0.01) A	SW 0.27 (0.04) 5	SW 0.35 (0.08) 5
		TP 0.04 (0.01) W	SW 0.37 (0.11) 6	TP 0.04 (0.01) W	SW 0.25 (0.04) 6	SW 0.28 (0.10) 6
			SW 0.33 (0.11) 7		SW 0.23 (0.04) 7	SW 0.21 (0.10) 7
			TP 0.70 (0.07) 1		TP 0.24 (0.05) 1	TP 0.57 (0.15) 1
			TP 0.66 (0.06) 2		TP 0.22 (0.04) 2	TP 0.49 (0.15) 2
			TP 0.61 (0.06) 3		TP 0.20 (0.04) 3	TP 0.41 (0.15) 3
			TP 0.57 (0.06) 4		TP 0.18 (0.03) 4	TP 0.34 (0.15) 4
			TP 0.52 (0.06) 5		TP 0.16 (0.03) 5	TP 0.27 (0.14) 5
			TP 0.48 (0.07) 6		TP 0.15 (0.03) 6	TP 0.21 (0.13) 6
			TP 0.43 (0.08) 7		TP 0.13 (0.03) 7	TP 0.16 (0.12) 7

Outputs are shown for models that were within two AIC of the most supported model. ‘s’ represents study site (SW = Southwest or TP = Tasman Peninsula), ‘h’ represents habitat (D = dry eucalypt forest and woodland, W = wet eucalypt forest and woodland, R = rainforest, A = agricultural and/or exotic vegetation), ‘b’ represents days since baiting (1 = less than one day since baiting, 2 = less than two days since baiting and so on) and ‘.’ represents constant. Numbers in brackets represent standard error.

- Swamp rats had a lower probability of site occupancy than devils, but they had a higher probability of detection.
- Patterns were inconsistent regarding the detection of swamp rats at sites where devils were detected: the probability of detection of swamp rats increased at sites where devils were detected in the Southwest according to one of the top models, but decreased according to the other top model. Swamp rat detection decreased at sites where devils were detected under both models for the Tasman Peninsula.
- The probability of detecting devils was unaffected by the detection of swamp rats.
- The two species were detected less often within the same 24 h period than expected if they were detected independently. Estimates of delta under the most supported models decreased with days since baiting at both sites and ranged from 0.66 (±0.06 SE) –0.16 (±0.08 SE) in the Southwest and 0.58 (±0.15 SE) –0.12 (±0.09 SE) on the Tasman Peninsula.
- Habitat and study site were important covariates for devils. Time since baiting, habitat and study site were important covariates for swamp rats.

#### Feral cats and swamp rats

Feral cat and swamp rat models within two AIC points of the top model (Table 5, Table 8) show that:

**Table 8:** Model outputs from two-species occupancy models for feral cats and swamp rats.

p*Fc* (bxs),p*Rl* (bxs+h),r*Fc* (bxs),r*Rl* (bxs+h),delta (s)						
Psi *Fc*	Psi *Rl*	p *Fc*	p *Rl*	r *Fc*	r *Rl*	delta
0.99 (0.15)	0.11 (0.06)	SW 0.10 (0.02) 1	SW 0.36 (0.09) D 1	SW 0.11 (0.03) 1	SW 0.57 (0.06) 1	SW 0.49 (0.06)
		SW 0.09 (0.02) 2	SW 0.30 (0.07) D 2	SW 0.09 (0.02) 2	SW 0.56 (0.06) 2	TP 0.68 (0.05)
		SW 0.09 (0.02) 3	SW 0.25 (0.06) D 3	SW 0.08 (0.02) 3	SW 0.54 (0.06) 3	
		SW 0.08 (0.01) 4	SW 0.20 (0.05) D 4	SW 0.07 (0.02) 4	SW 0.52 (0.06) 4	
		SW 0.07 (0.01) 5	SW 0.16 (0.05) D 5	SW 0.06 (0.02) 5	SW 0.50 (0.06) 5	
		SW 0.07 (0.02) 6	SW 0.13 (0.04) D 6	SW 0.05 (0.02) 6	SW 0.49 (0.06) 6	
		SW 0.07 (0.02) 7	SW 0.10 (0.04) D 7	SW 0.05 (0.02) 7	SW 0.47 (0.07) 7	
		TP 0.19 (0.03) 1	TP 0.78 (0.06) D 1	TP 0.07 (0.02) 1	TP 0.34 (0.06) D 1	
		TP 0.17 (0.03) 2	TP 0.73 (0.06) D 2	TP 0.07 (0.02) 2	TP 0.33 (0.05) D 2	
		TP 0.16 (0.02) 3	TP 0.67 (0.06) D 3	TP 0.06 (0.01) 3	TP 0.31 (0.05) D 3	
		TP 0.15 (0.02) 4	TP 0.61 (0.06) D 4	TP 0.05 (0.01) 4	TP 0.30 (0.04) D 4	
		TP 0.14 (0.02) 5	TP 0.54 (0.07) D 5	TP 0.04 (0.01) 5	TP 0.28 (0.04) D 5	
		TP 0.13 (0.02) 6	TP 0.48 (0.08) D 6	TP 0.04 (0.01) 6	TP 0.27 (0.05) D 6	
		TP 0.12 (0.03) 7	TP 0.41 (0.09) D 7	TP 0.03 (0.01) 7	TP 0.26 (0.05) D 7	
**p** ***Fc*** ** (bxs),p** ***Rl*** ** (bxs+h), r** ***Fc*** ** (bxs),r** ***Rl*** ** (bxs+h),delta(bxs)**						
0.99 (0.15)	0.11 (0.06)	SW 0.10 (0.02) 1	SW 0.36 (0.09) D 1	SW 0.10 (0.03) 1	SW 0.57 (0.06) 1	SW 0.49 (0.08) 1
		SW 0.09 (0.02) 2	SW 0.30 (0.07) D 2	SW 0.09 (0.02) 2	SW 0.56 (0.06) 2	SW 0.49 (0.07) 2
		SW 0.09 (0.02) 3	SW 0.25 (0.06) D 3	SW 0.08 (0.02) 3	SW 0.54 (0.06) 3	SW 0.49 (0.06) 3
		SW 0.08 (0.01) 4	SW 0.20 (0.05) D 4	SW 0.07 (0.02) 4	SW 0.52 (0.06) 4	SW 0.49 (0.06) 4
		SW 0.07 (0.01) 5	SW 0.16 (0.05) D 5	SW 0.06 (0.02) 5	SW 0.50 (0.06) 5	SW 0.49 (0.08) 5
		SW 0.07 (0.02) 6	SW 0.13 (0.04) D 6	SW 0.05 (0.02) 6	SW 0.49 (0.06) 6	SW 0.49 (0.09) 6
		SW 0.06 (0.02) 7	SW 0.10 (0.04) D 7	SW 0.05 (0.02) 7	SW 0.47 (0.07) 7	SW 0.49 (0.11) 7
		TP 0.19 (0.03) 1	TP 0.78 (0.06) D 1	TP 0.08 (0.02) 1	TP 0.34 (0.06) D 1	TP 0.67 (0.06) 1
		TP 0.17 (0.02) 2	TP 0.73 (0.06) D 2	TP 0.07 (0.02) 2	TP 0.33 (0.05) D 2	TP 0.67 (0.07) 2
		TP 0.16 (0.02) 3	TP 0.67 (0.06) D 3	TP 0.06 (0.01) 3	TP 0.31 (0.05) D 3	TP 0.68 (0.05) 3
		TP 0.15 (0.02) 4	TP 0.61 (0.06) D 4	TP 0.05 (0.01) 4	TP 0.30 (0.04) D 4	TP 0.68 (0.05) 4
		TP 0.14 (0.02) 5	TP 0.54 (0.07) D 5	TP 0.04 (0.01) 5	TP 0.28 (0.04) D 5	TP 0.68 (0.07) 5
		TP 0.13 (0.02) 6	TP 0.48 (0.08) D 6	TP 0.04 (0.01) 6	TP 0.27 (0.05) D 6	TP 0.68 (0.08) 6
		TP 0.12 (0.03) 7	TP 0.41 (0.09) D 7	TP 0.03 (0.01) 7	TP 0.26 (0.05) D 7	TP 0.68 (0.10) 7

Outputs are shown for models that were within two AIC of the most supported model. ‘s’ represents study site (SW = Southwest or TP = Tasman Peninsula), ‘h’ represents habitat (D = dry eucalypt forest and woodland, W = wet eucalypt forest and woodland, R = rainforest, A = agricultural and/or exotic vegetation), ‘b’ represents days since baiting (1 = less than one day since baiting, 2 = less than two days since baiting and so on) and ‘.’ represents constant. Numbers in brackets represent standard error.

- Swamp rats had a lower probability of site occupancy than feral cats, but swamp rats had a higher probability of detection.
- The probability of detecting feral cats at cameras where swamp rats were present was similar for the Southwest, but reduced on the Tasman Peninsula.
- The probability of detecting swamp rats at sites where feral cats were detected was greater in the Southwest but less on the Tasman Peninsula compared to sites where feral cats were not detected.
- The two species were detected less often within the same 24 h period than expected if they were detected independently. Estimates of delta were 0.49 (±0.06 SE) for the Southwest and 0.67–0.68 (±0.05 SE) for the Tasman Peninsula.
- Time since baiting, habitat and study site were important covariates for swamp rats. Time since baiting and study site were important covariates for feral cats.

### Small Mammal Trap Responses to Predator Odours

**Figure 7:**
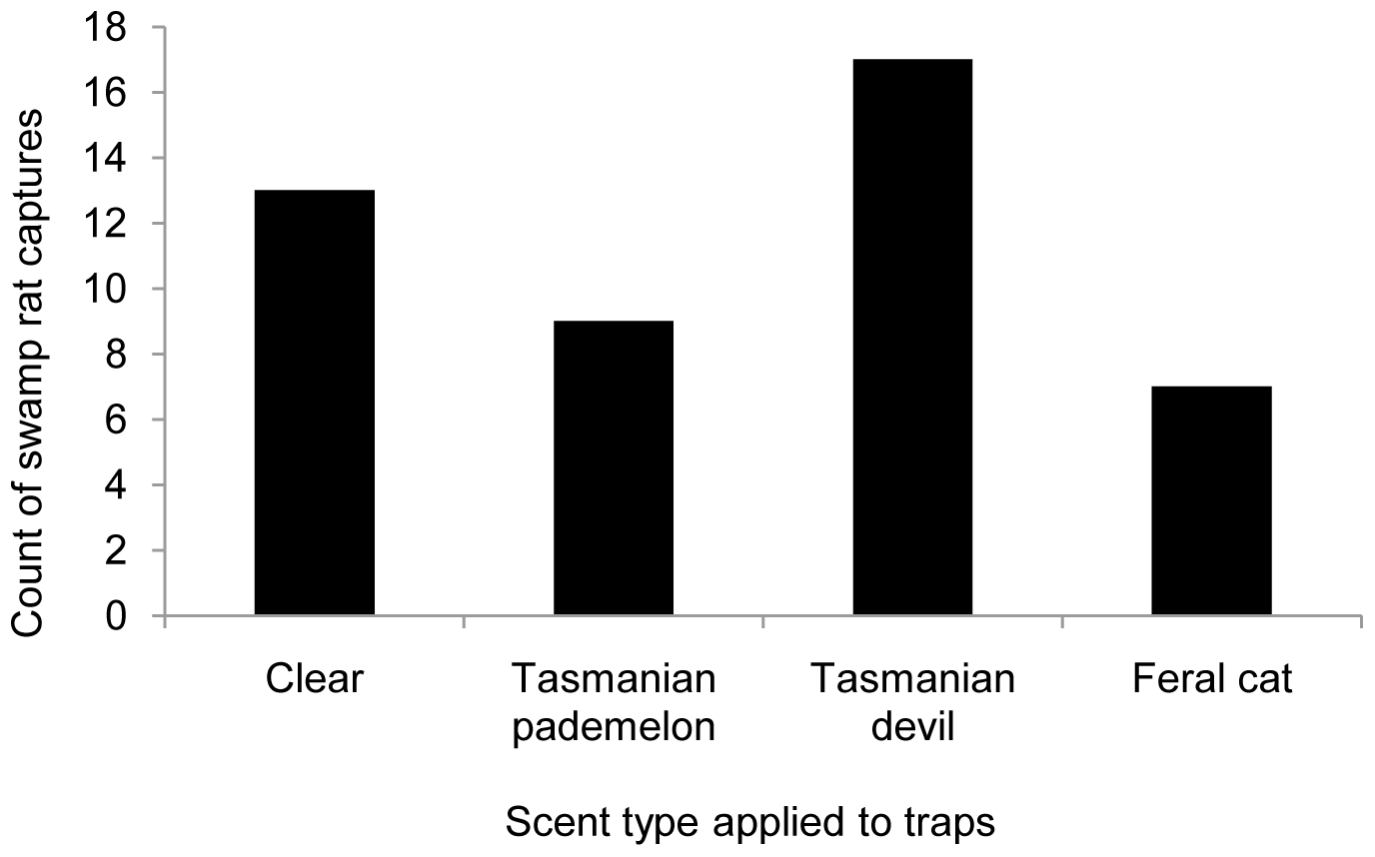
Captures (*n* = 46) of swamp rat *Rattus lutreolus* in Elliott traps scented with different faecal odours. Traps were scented with one of four different odours: clear (no faecal smear), Tasmanian pademelon *Thylogale billardierii* faeces, Tasmanian devil *Sarcophilus harrisii* faeces and feral cat *Felis catus* faeces.

There were 46 captures of swamp rats across the two study sites (Table 9) and fewer were captured in traps scented with feral cat odour compared to the other odours (Fig. 7). In comparison, devil-scented traps captured the most swamp rats compared to other odours. While trends were apparent, there were no significant differences in response to odour by swamp rats (χ^2^_3_ = 5.13, *p* = 0.162).

**Table 9:** Swamp Rat captures in scented traps.

Site	Survey	Trap nights	Clear	Tasmanian Pademelon	Tasmanian devil	Feral cat
Tasman Peninsula	April 2011	560	8	2	5	2
Mt Field	June 2011	480	5	7	12	5

The number of captures of the Swamp Rat *Rattus lutreolus* for each survey at two study sites in Elliott traps scented with clear (no faecal smear), Tasmanian Pademelon *Thylogale billardierii* faeces, Tasmanian Devil *Sarcophilus harrisii* faeces, and feral cat faeces. Trap nights are expressed as number of traps multiplied by nights set.

## Discussion

Fear, manifested as avoidance behaviour, can be an important factor in shaping individual behaviour and population dynamics [23], and can be interpreted as a survival adaptation. Fear responses have been observed in a wide range of taxa, including mesopredators and prey [17]. A better understanding of avoidance behaviour is a precursor to effective conservation management and to understanding ecological interactions more generally. This is especially important for invasive predators because they often have particularly adverse impacts on prey [43], [44]. In addition, mesopredator relationships between invasive predators can lead to unexpected outcomes for other species in the absence of an integrated pest management approach [45]. We investigated patterns of detection between two predators, the endangered marsupial Tasmanian devil and the introduced eutherian feral cat, and one native small mammal, the swamp rat.

We found that feral cats were detected less often at cameras where devils were detected; this was apparent at all three of our study sites, and occurred for the duration of the 1–2 week study site surveys. Devils also tended to be detected more often at cameras where feral cats had been detected. The responses of the swamp rat to devil and cat cues at cameras were less clear and depended on study site, although capture rates of swamp rats in Elliott traps tended to be less where feral cat but not devil faecal odours were present. Thus, the results of the devil and feral cat two-species occupancy analyses at cameras supported our initial hypothesis, but models incorporating the swamp rat were less clear. The results of the faecal odour trials provided very limited support for our hypothesis that swamp rats would avoid feral cat scented traps, and no support for our hypothesis that they would avoid devil scented traps.

### Two-Species Occupancy Models

We used a series of two-species occupancy models to elucidate patterns of detection between the three study species. Our models showed that the probability of detecting a given species could be associated with the detection of two or more species, thus indicating that patterns of detection are at least three-way, and realistically might be far more complex given that over 20 species were recorded at cameras. In this regard our models were a considerable simplification of the real system. Modelling interactions among multiple species within a maximum likelihood framework is not yet possible with available software. A three-species model for one predator and two prey has been developed [46]; however, this model does not accommodate two predators and one prey, or hierarchical interactions between two predators such as that implied in the present study between devils and feral cats.

Despite the potential influences of multiple species, the consistent pattern of detection between devils and feral cats in three study sites is compelling evidence that devils can alter feral cat detection. There have been suggestions of a mesopredator relationship between devils and cats following observations of a marked increase in spotlight counts and anecdotal reports of feral cats in the north-east of Tasmania in recent years (Department of Primary Industries and Water, unpublished data) [37]. This latter region is where DFTD appears to have been longest established and where devil populations have suffered the most dramatic declines to date. Whether these observations truly represent mesopredator release is yet to be determined. It is also important to note that if there is a competitive relationship between devils and feral cats, the strengths and characteristics of this relationship may vary spatially and temporally. For example, at higher abundances feral cats may overwhelm prey resources or kill significant numbers of juvenile devils [47]. Also, preferential use of trails has been recorded in carnivore species in sand dune habitats, and we might have recorded different patterns of detection if we had set cameras randomly throughout the landscape rather than limiting them to roads and trails [48].

Aspects of devil biology that may have facilitated their apparent dominance at baited camera stations include efficient scavenging abilities [49] and scent-marking. We regularly recorded devils scent marking camera stations (via anal-dragging, urination and defecation), whereas none of the other species photographed showed such obvious behaviours consistent with scent marking. The function of scent marking in devils is unknown, but it is reasonable to expect that it serves as a signal to other animals, including feral cats, that a devil may return. Most mammals scent mark [50] and it is believed to play an important role in inter- and intra-specific communication [25], [51].

Patterns of detection between swamp rats, feral cats and devils were not as consistent as those between devils and feral cats. This inconsistency may result from inadequate model structure: as noted previously, a series of two-species models was used to represent a three-way system and we were not able to fully incorporate season as a covariate in our models due to the large number of parameters involved. Single species multi-season site occupancy models indicated that there was some seasonal variability in the probability of detecting swamp rats [38]. Alternatively, the patterns of swamp rat detection that we observed may reflect reality, with animals differing in detection in different areas. Biological factors that could underlie such differences include competition between conspecifics [5], [52] different predator abundances and therefore prey vigilance [53], and different risk thresholds associated with factors such as food availability or fine-scale habitat preferences [23], [52].

### Faecal Odour Trials

Faecal odour trials suggested a tendency for swamp rats to be captured less often in feral cat scented traps and more often in devil scented traps, but these trends were not significant. It is possible that our study may not have been powerful enough to detect real avoidance. If our findings were a true reflection of reality, however, then they provide partial support for a study that found swamp rats in the laboratory avoided integumental odour (odour trapped in hair from secretions of sebaceous glands on the skin) of the spotted-tailed quoll (a native carnivore in Tasmania), but did not avoid integumental odour from the red fox or feral cat [54]. Differential avoidance of odours highlights the potential for variability in measures of predator recognition depending on the predator and type of cue.

If strength of avoidance of predator cues is proportional to the intensity or duration of selection [28], feral cats could be expected to exert more predatory pressure on swamp rats than devils. In the first instance, small mammals occur much less frequently in the diet of the devil than do medium-sized mammals [55], [56], [57]. Moreover, the frequency of occurrence of small mammals is much lower in the diet of the devil compared to that in the diet of feral cats [38]. If this reasoning is correct, our failure to find stronger avoidance by swamp rats of the odour of feral cats is surprising and may suggest that selection has not been as intense as we had expected. This could reflect insufficient time for selection to have acted or, perhaps more plausibly, that devils maintained cats at such low densities prior to the arrival of DFTD that their predatory impacts on swamp rats were usually weak. Conversely, faeces may not represent reliable cues to the current risk of predation; integumental odours may arguably be more representative of impending attack and may therefore induce different and perhaps more marked anti-predator behaviour [54]. In addition, predator cues are not limited to odour, they can also include visual and acoustic cues [58], [59].

We hesitate to compare the responses by swamp rats to the predator activity recorded at cameras, and the odour cues on traps, for three reasons: there was, firstly, an inconsistent response by swamp rats at cameras; secondly, our two-species models may not represent reality to the degree necessary to reliably quantify avoidance; and thirdly, the responses of rats to odours were weak. The potential for prey avoidance to vary with cue, study site, season, and habitat structure highlights the need to conduct predator avoidance studies that account for these factors [60].

## Conclusions

Patterns of detection at cameras of devils and feral cats are consistent with the interpretation of a dominant predator – mesopredator relationship, and warrant further investigation given that devils have declined dramatically as a result of devil facial tumour disease. Because small mammals constitute a higher proportion of the diet of feral cats compared with devils, there may be an increased risk to populations of small mammals if there is an increase in the activity and thus impact of feral cats resulting from devil decline. Feral cat impacts may be exacerbated further if swamp rats truly exhibit weak avoidance of predator scents. Further investigation could focus profitably on the responses of other small Tasmanian mammals to cat cues and cat predation, especially scarce endemic species that could be at particularly increased risk. More generally, we suggest that population estimates derived from devices such as remote cameras should acknowledge the potential for one species to change the detectability of another, and ensure that this potential bias is incorporated in assessments of numbers and survival.
